# WITHDRAWN: TRIM44 promotes autophagy through SQSTM1 oligomerization in the response to oxidative stress induced by Arsenic Trioxide in cancer cells

**DOI:** 10.21203/rs.3.rs-3951960/v1

**Published:** 2024-02-20

**Authors:** Yuqin Wang, Lin Lyu, Trung Vu, Nami McCarty

**Affiliations:** University of Texas Medical School

## Abstract

The authors have requested that this preprint be removed from Research Square.

